# Relationship between Sleep Duration, Sun Exposure, and Serum 25-Hydroxyvitamin D Status: A Cross-sectional Study

**DOI:** 10.1038/s41598-020-61061-8

**Published:** 2020-03-06

**Authors:** Ji Ho Choi, Bora Lee, Jae Yong Lee, Chang-Hoon Kim, Bumhee Park, Dong Young Kim, Hyun Jun Kim, Do-Yang Park

**Affiliations:** 1 Department of Otorhinolaryngology-Head and Neck Surgery, Soonchunhyang University College of Medicine, Bucheon Hospital, Bucheon, Republic of Korea; 2 Department of Statistics, Graduate School of Chung-Ang University, Seoul, Republic of Korea; 3 Department of Otorhinolaryngology, Yonsei University College of Medicine, Seoul, Republic of Korea; 4 Department of Biomedical Informatics, Ajou University School of Medicine, Suwon, Republic of Korea; 5 Office of Biostatistics, Ajou Research Institute for Innovative Medicine, Ajou University Medical Center, Suwon, Republic of Korea; 6 Department of Otolaryngology, Ajou University School of Medicine, Suwon, Republic of Korea; 7 Department of Medicine, Yonsei University Graduate School, Seoul, Republic of Korea

**Keywords:** Epidemiology, Preclinical research, Sleep disorders, Risk factors

## Abstract

Normal-range sleep duration is an important factor for general health and metabolism, and insufficient or excessive sleep is associated with chronic metabolic disease. Among the many factors that affect sleep duration, sun exposure plays an important role in maintaining regular circadian rhythm and is also involved in the production and activation of 25-hydroxyvitamin D [25(OH)D], which regulates various functions in the body. However, 25(OH)D is available through food and various nutritional supplements without sun exposure, so it is important to find out the complex relationship among sun exposure, vitamin D status, and sleep duration. The relationship between sun exposure, vitamin D status, and sleep duration was analyzed in the nationwide survey and examination of 25,534 study populations, after adjusting for demographic characteristics, physical characteristics, lifestyle status, and socio-demographic variables. Vitamin D status alone did not show the relationship with sleep duration, although there were statistical relationships in the various factors including sun exposure with sleep duration. There was a statistical difference in 25(OH)D according to sleep duration, only in low sun exposure group. Subjects with low sun exposure and excessive sleep duration comparatively lower 25(OH)D than those with normal-range sleep, even after adjustment for potentially confounding factors. Individuals with limited exposure to sunlight should maintain adequate vitamin D status to have an appropriate sleep duration for health.

## Introduction

Normal-range sleep duration is an important factor for general health and metabolism^1^. Insufficient or excessive sleep duration is associated with various chronic physical or mental illness, such as obesity, hypertension, diabetes, metabolic syndrome, depression, and other psychiatric disease^2–4^. Sleep insufficiency has been also associated with low thyroid hormone levels in rats and with high noradrenaline and cortisol levels in humans^5^. Sleep duration is mainly affected by circadian rhythm, and circadian rhythm is affected by the melatonin level controlled by the light exposure^6–8^. Therefore, sun exposure has an important role for determining sleep time^9–11^.

25-hydroxyvitamin D [25(OH)D], which is also greatly affected by sunlight exposure, regulates the metabolism of calcium and phosphorus, which contribute to the maintenance of a healthy musculoskeletal system. Recent studies have suggested that vitamin D status are associated with impaired glucose metabolism, cardiovascular disease, infectious/inflammatory disease, psychiatric disease, and cancer^12–16^. 25(OH)D is produced either through an endogenous pathway using UV light from sun exposure or an exogenous pathway through food intake. Vitamin D status are mainly regulated through serum phosphorus and parathyroid hormone, but are also affected by temperature, skin color, sunscreen use, clothing, obesity, and hepatic or renal function^17^. Serum vitamin D status, measured as 25(OH)D, are also implicated in an increasing number of physiological mechanisms, including sleep^18^. The 25(OH)D receptor is widely distributed among tissues, including the regions of the brain involved in sleep regulation and central nervous system inflammatory signaling^19–21^. The circadian phase of sleep can be delayed by vitamin D supplementation and by sun exposure^22,23^. Finally, serum vitamin D status have been related to daytime sleepiness^24^, and epidemiologic studies have indicated that vitamin D status are associated with the mid-point of sleep, sustained sleep, and sleep duration in the elderly^25–27^.

Modern human life often results in insufficient sun exposure, so it is important that 25(OH)D studies consider this factor. The complex metabolic process of production and activation of 25(OH)D by sun exposure, and the fact that 25(OH)D can be supplemented by exogenous methods, such as food or nutritional supplementation, without endogenous process of sun exposure implies that various related factors, including sun exposure, must be considered in analyzing the relationships between sleep duration and 25(OH)D. Therefore, we designed our study to analyze the relationship between vitamin D status, sleep duration, and sun exposure after controlling for a number of potentially confounding variables.

## Methods

### Study population

The Korean National Health and Nutrition Examination Survey (KNHANES) is a nationwide survey conducted by the Korea Centers for Disease Control and Prevention, in conjunction with the Korean Society of Otorhinolaryngology-Head and Neck Surgery (KORL-HNS) and other societies. KNHANES was initiated in 1998 to record the health and nutritional status of the Korean population and was designed as a multistage, cross-sectional, stratified sampling study without overlapping subjects. Teams of four medical experts, including an otolaryngologist, conducted the clinical examinations nationwide, using a specially equipped mobile examination vehicle. All questionnaires were completed, samples taken, and examinations performed for each subject in a single visit. KORL-HNS trained the survey teams to standardize the examinations. Our study was conducted on KNHANES data obtained between 2010 and 2012 (n = 25534). A total of 14490 participants were included in the final study population after the exclusion of participants aged less than 19 years (n = 5,935) and those with missing relevant data (n = 5,109). The mean age of study participants was 50.84 ± 16.26 years (range, 19–97 years) and the male to female ratio was 1:1.47. The survey protocol was approved by the institutional review board of the Korea Centers for Disease Control and Prevention (IRB Nos. 2010-02CON-21-C, 2011-02CON-06-C, and 201201EXP-01-2C). All study participants provided written informed consent as part of KNHANES.

### Assessment of sleep duration and sun exposure

Self-reported sleep duration was assessed through a questionnaire. A KNHANES nurse asked survey participants “How many hours a day do you usually sleep?” and recorded their response^28^. From this response, sleep duration was divided into three groups: less than 6 hours, 6 to 9 hours, and more than 10 hours. To assess daily sun exposure, the participants were asked, “What is the average duration of your direct exposure to sun during the day?” for which participants were given the options: less than 2 hours, 2 to 5 hours, and 5 hours or more.

### Measurement of serum 25-hydroxy vitamin D status

Blood samples were collected via the antecubital vein the morning after a minimum fast of 8 hours and refrigerated immediately. Samples were transported to the central testing facility in cold storage and analyzed within 24 h. Serum levels of 25(OH)D were measured by radioimmunoassay (25(OH)D ^125^I RIA Kit; DiaSorin, Still Water, MN) using a gamma-counter (1470 Wizard; PerkinElmer, Turku, Finland). The inter assay coefficients of variation were 11.7%, 10.5%, and 8.6% at 21.47, 56.66, and 82.37 nmol/L, respectively. KNHANES participates in the Vitamin D Standardization Program, so the measurement of 25(OH)D was standardized with the Belgian National Institute of Standards and Technology reference procedure recently developed by Ghent University^29^. Although there has been much debate over the definition of vitamin D deficiency, it is generally accepted that a 25(OH)D concentration of 20 ng/mL is an indication of deficiency^30–32^.

### Statistical analysis

Statistical analyses were performed using the SAS survey procedure (ver. 9.4; SAS Institute, Cary, NC, USA) because of the complex sampling design and sampling weights from KNHANES. The procedure accommodated unequal probabilities of selection, oversampling, and non-response. Participants’ characteristics were analyzed using mean and standard error for continuous variables and value and percentage for categorical variables. To analyze differences in serum vitamin D status between sleep duration groups, the Rao-Scott chi-square test (using PROC SURVEYFREQ in SAS) was used. The exponentiated beta coefficients and 95% confidence intervals (CIs) for log-transformed vitamin D status were calculated.

Multiple logistic regression analysis (using PROC SURVEYLOGISTIC in SAS) was used to determine the association between 25(OH)D, sun exposure, and sleep duration. The crude dataset (model 1) was adjusted for age and sex (model 2). Model 2 was then adjusted for physical status (obesity, prevalent hypertension [HTN], prevalent diabetes [DM], and prevalent dyslipidemia) to generate model 3. Finally, model 3 was adjusted for lifestyle status (smoking status, drinking status, and regular exercise) and socio-demographic factors (family income, educational level, occupation, and region of residence), generating model 4. Two-tailed p values were generated, and a p < 0.05 was considered significant.

### Ethical approval

All procedures performed in the studies involving human participants were in accordance with the ethical standards of the institutional and/or national research committee and with the 1964 Helsinki declaration and its later amendments or comparable ethical standards.

### Informed consent

Informed consent was obtained from all individual participants included in the study.

## Results

### Baseline characteristics and associations with sleep duration

A total of 14490 enrolled participants were divided into three groups according to sleep duration: normal-range sleep group (‘6 to 9 hours’, 83.6%), sleep insufficient group (‘less than 6 hours’, 13%), and excessive sleep group (‘more than 10 hours’, 3.4%). Data were analyzed to identify statistical differences between groups in terms of age, sex, obesity, HTN, DM, dyslipidemia, smoking status, alcohol consumption, regular exercise, region of residence, family income, education level, occupation, exposure to sunlight, and vitamin D status (Table 1). All factors differed between groups apart from regular exercise and vitamin D status. In the excessive sleep group, participants were more likely to be younger, non-hypertensive, non-dyslipidemic, and menstruating women. In the sleep insufficient group, participants tended to be older, overweight, relatively hypotensive, DM, dyslipidemic, non-smokers, non-alcohol drinkers, and menopausal women. The percentage of residents living in urban areas was higher in the sleep insufficient group than in the excessive sleep group. Participants with low sun exposure were most likely to be in the normal-range sleep group than in the sleep insufficient or excessive sleep group. Vitamin D status were not significantly different between sleep duration groups prior to adjustment for potentially confounding factors.

**Table 1:** Baseline characteristics by sleep duration.

Variable			Sleep duration (hours/day)			
		N	<6	6 to 9	≥10	p-value
N			2226 (13%)	11798 (84%)	466 (3%)	
Age (years)	19–29	1586	10.6 (1)	20 (0.7)	39.5 (1)	**<0.01**
	30–39	2612	11.2 (1)	22.6 (0.7)	10.4 (2)	
	40–49	2556	19.3 (1)	22.9 (0.6)	13.7 (2)	
	50–59	2867	20.3 (1)	18.5 (0.5)	15.4 (2)	
	60–69	2609	18 (1)	9.5 (0.3)	10.7 (1)	
	≥70	2260	20.5 (1)	6.4 (0.3)	10.3 (1)	
Sex	Male	5869	40.9 (1)	51.6 (0.5)	50.4 (3)	**<0.01**
	Menstruating women	4271	21.1 (1)	31.6 (0.5)	34.9 (3)	
	Menopausal women	4350	38 (1)	16.8 (0.4)	14.8 (2)	
Obesity	Underweight	634	2.9 (0.4)	4.8 (0.3)	6.9 (2)	**<0.01**
	Normal	9234	62.3 (1)	63.4 (0.6)	62.6 (3)	
	Overweight	4585	34.8 (1)	31.8 (0.6)	30.5 (3)	
Hypertension	No	11043	72.3 (1)	84.4 (0.5)	85.1 (2)	**<0.01**
	Yes	3447	27.7 (1)	15.6 (0.5)	14.9 (2)	
Diabetes	No	13241	90.2 (0.7)	94.2 (0.3)	93.8 (1)	**<0.01**
	Yes	1249	9.8 (0.7)	5.8 (0.3)	6.2 (1)	
Dyslipidemia	No	12798	87 (1)	92.1 (0.3)	93.2 (1)	**<0.01**
	Yes	1692	13 (1)	7.9 (0.3)	6.8 (1)	
Smoking status	Non-smoker	8829	60.2 (1)	53 (0.5)	48.2 (3)	**<0.01**
	Ex-smoker	2967	18.7 (1)	20.9 (0.5)	20.5 (2)	
	Smoker	2694	21.1 (1)	26.1 (0.6)	31.3 (3)	
Alcohol consumption	None	4186	32.4 (1)	20.8 (0.5)	25.2 (3)	**<0.01**
	Occasional (<2/week)	7416	45.7 (1)	56.0 (0.6)	50.3 (3)	
	Frequent (≥2/week)	2888	21.9 (1)	23.2 (0.5)	24.5 (3)	
Regular exercise	No	7694	51.1 (2)	51.4 (0.7)	55.3 (3)	0.35
	Yes	6796	48.9 (2)	48.6 (0.7)	44.7 (3)	
Region of residence	Rural	4844	30.4 (2)	29.1 (1)	36.9 (3)	**0.01**
	Urban	9646	69.6 (2)	70.9 (1)	63.1 (3)	
Family income	<50^th^ percentile	6573	52.9 (2)	40.5 (1)	52.7 (3)	**<0.01**
	≥50^th^ percentile	7917	47.1 (2)	59.5 (1)	47.3 (3)	
Educational level	Middle school or lower	5397	48.3 (1)	24.4 (0.7)	33.8 (3)	**<0.01**
	High school or higher	9093	51.7 (1)	75.6 (0.7)	66.2 (3)	
Occupation	White collar	4604	27.1 (1)	39.7 (0.7)	24.7 (3)	**<0.01**
	Blue collar	3887	29.7 (1)	27.4 (1)	23.7 (3)	
	Unemployed	5999	43.1 (1)	32.9 (0.6)	51.6 (3)	
Exposure to sunlight (hours/day)	<2	9173	59.2 (2)	63.5 (1)	58.1 (3)	**<0.01**
	≥2	5317	40.8 (2)	36.5 (1)	41.9 (3)	
25(OH)D (ng/mL)	<20	10217	71.1 (2)	71.7 (1)	74.4 (3)	0.48
	≥20	4273	28.9 (2)	28.3 (1)	25.6 (3)	

Data were reported as weighted percentage (SE). p-Values were calculated by Rao-Scott chi-square test.

### Association of vitamin D status with baseline characteristics and association of sleep duration with vitamin D sufficiency/deficiency

Vitamin D status in participants were analyzed with respect to age, sex, obesity, HTN, DM, dyslipidemia, smoking status, alcohol consumption, regular exercise, region of residence, family income, education level, occupation, and exposure to sunlight. Participants were also divided according to vitamin D status into a deficiency group (<20 ng/mL, 71.74%) and a sufficiency group (≥20 ng/mL, 28.26%), and groups were analyzed for an association with baseline characteristics (Table 2). Vitamin D status were higher in participants who were elderly, overweight, hypertensive, diabetic, and dyslipidemic and lower in menstruating women. Participants with appropriate vitamin D status were more likely to participate in regular exercise, live in a rural area, have a low familial income and educational level, be blue collar workers, and have adequate sun exposure. Vitamin D status were relatively high in the participants with sleep insufficiency.

**Table 2:** Vitamin D status by participant characteristics.

Variable			25(OH)D (ng/mL)	25(OH)D < 20 ng/mL	25(OH)D ≥ 20 ng/mL	
		N	Geometric mean (SE)	% (SE)	% (SE)	p-value
Total			16.3 (1)	71.7 (1)	28.3 (1)	
Age (years)	19–29	1586	14.5 (1)	22.8 (0.7)	11 (1)	**<0.01**
	30–39	2612	15.6 (1)	22.5 (0.7)	16.3 (1)	
	40–49	2556	16.3 (1)	22.3 (0.6)	21.6 (1)	
	50–59	2867	17.6 (1)	16.7 (0.5)	23.5 (1)	
	60–69	2609	18.2 (1)	8.7 (0.3)	15.6 (0.7)	
	≥70	2260	17.9 (1)	7 (0.3)	11.9 (0.6)	
Sex	Male	5869	17.3 (1)	45.9 (0.6)	60.8 (1)	**<0.01**
	Menstruating women	4271	14.5 (1)	35.7 (0.6)	16.9 (1)	
	Menopausal women	4350	17.1 (1)	18.4 (0.5)	22.3 (1)	
Obesity	Underweight	634	14.9 (1)	5 (0.3)	3.5 (0.4)	**<0.01**
	Normal	9234	16.3 (1)	63.3 (0.6)	63.2 (1)	
	Overweight	4585	16.6 (1)	31.7 (0.7)	33.3 (1)	
Hypertension	No	11043	16.1 (1)	84.6 (0.5)	78.3 (1)	**<0.01**
	Yes	3447	17.5 (1)	15.4 (0.5)	21.7 (1)	
Diabetes	No	13241	16.2 (1)	94.5 (0.3)	91.5 (0.5)	**<0.01**
	Yes	1249	17.6 (1)	5.5 (0.3)	8.5 (0.5)	
Dyslipidemia	No	12798	16.2 (1)	92.2 (0.3)	89.4 (0.5)	**<0.01**
	Yes	1692	17.4 (1)	7.8 (0.3)	10.6 (0.5)	
Smoking status	Non-smoker	8829	15.8 (1)	56.9 (0.6)	45.9 (1)	**<0.01**
	Ex-smoker	2967	17.6 (1)	18.1 (0.5)	27.2 (1)	
	Smoker	2694	16.5 (1)	25.1 (0.6)	26.9 (1)	
Alcohol consumption	None	4186	16.2 (1)	22.5 (0.6)	22.2 (1)	**<0.01**
	Occasional (**<**2/week)	7416	15.9 (1)	56.7 (0.7)	49.1 (1)	
	Frequent (≥2/week)	2888	17.5 (1)	20.9 (0.6)	28.7 (1)	
Regular exercise	No	7694	16.1 (1)	52.8 (0.7)	48.3 (1)	**<0.01**
	Yes	6796	16.6 (1)	47.2 (0.7)	51.7 (1)	
Region of residence	Rural	4844	17.8 (1)	25.2 (1)	40.7 (2)	**<0.01**
	Urban	9646	15.7 (1)	74.8 (1)	59.3 (2)	
Family income	**<**50^th^ percentile	6573	16.6 (1)	41 (1)	46.3 (1)	**<0.01**
	≥50^th^ percentile	7917	16.2 (1)	59 (1)	53.7 (1)	
Educational level	Middle school or lower	5397	17.8 (1)	23.8 (0.7)	37.9 (1)	**<0.01**
	High school or higher	9093	15.8 (1)	76.2 (0.7)	62.1 (1)	
Occupation	White collar	4604	15.8 (1)	40.4 (1)	30.5 (1)	**<0.01**
	Blue collar	3887	17.8 (1)	23.3 (0.7)	38.4 (1)	
	Unemployed	5999	15.8 (1)	36.3 (0.6)	31.2 (1)	
Sleep duration (hours/day)	**<**6	2226	16.5 (1)	12.9 (0.4)	13.3 (0.7)	0.48
	6 to 9	11798	16.3 (1)	83.5 (0.5)	83.6 (0.7)	
	≥10	466	15.7 (1)	3.6 (0.2)	3.1 (0.3)	
Exposure to sunlight (hours/day)	**<**2	9173	15.8 (1)	66.4 (1)	53.5 (1)	**<0.01**
	≥2	5317	17.3 (1)	33.6 (1)	46.5 (1)	

p-Values were calculated by Rao-Scott chi-square test.

The vitamin D deficiency and sufficiency groups showed significant differences in terms of age, sex, obesity, HTN, DM, dyslipidemia, smoking status, alcohol consumption, regular exercise, region of residence, family income, education level, occupation, and exposure to sunlight. However, no significant difference was observed between 25(OH)D groups in terms of sleep duration.

### Multiple logistic regression analyses of vitamin D status, sleep duration, and exposure to sunlight

**Figure 1:**
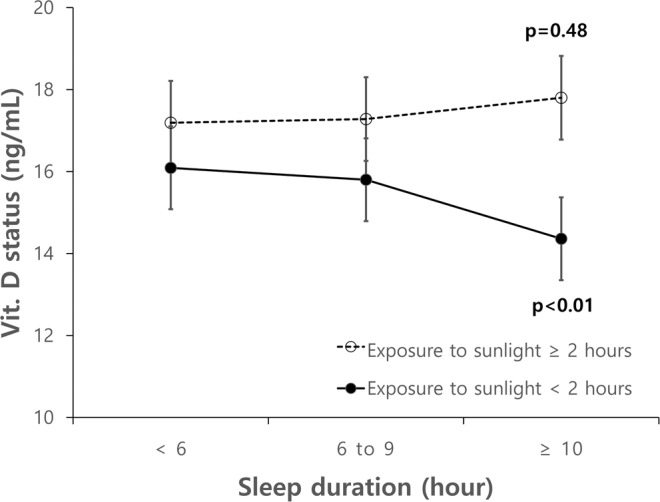
Distribution of Vit. D status.

Multiple logistic regression analyses were performed because of the conflicting evidence that there was no significant difference between the sleep duration groups and sun exposure, but there was a statistical difference in sleep duration within participants with low sun exposure (Table 3 and Fig. 1).

**Table 3:** Vitamin D status by sleep duration and sun exposure.

Variable			Sleep duration (hours/day)			
		N	<6 (N = 2226)	6 to 9 (N = 11798)	≥10 (N = 466)	p-value
Exposure to sunlight (hours/day)	<2	9173	16.1 (1)	15.8 (1)	14.4 (1)	**<0.01**
	≥2	5317	17.2 (1)	17.3 (1)	17.8 (1)	0.48

Data were reported as geometric mean (standard error, SE).

p-Values were calculated by analysis of variance.

To accurately assess the relationship between sleep duration, sun exposure, and vitamin D status, confounding factors of vitamin D status and sleep duration identified during this study were controlled through multiple logistic regression analysis. Differences in log-transformed vitamin D status between the sleep insufficient or excessive sleep groups and the normal-range sleep group were not significant. However, vitamin D status were significantly lower in the low exposure to sunlight group than in the adequate exposure to sunlight group, even after controlling for confounding variables (β = 0.91) (Table 4).

**Table 4:** Exponentiated beta coefficients and 95% confidence intervals of log-transformed vitamin D status.

Independent variable		Model 1		Model 2		Model 3		Model 4	
		β (95% CI)	p-value	β (95% CI)	p-value	β (95% CI)	p-value	β (95% CI)	p-value
Sleep duration (hour)	<6	0.99 (1)	0.74	0.97 (1)	0.06	0.97 (1)	0.06	0.97 (1)	0.07
	6 to 9	1 (Ref)		1 (Ref)		1 (Ref)		1 (Ref)	
	≥10	1.03 (1)	0.27	1.04 (1)	0.17	1.04 (1)	0.17	1.03 (1)	0.26
Exposure to sunlight (hour)	<2	0.91 (1)	**<0.01**	0.94 (1)	**<0.01**	0.94 (1)	**<0.01**	0.96 (1)	**<0.01**
	≥2	1 (Ref)		1 (Ref)		1 (Ref)		1 (Ref)	
Interaction terms	ETS < 2 & sleep duration < 6	1.02 (1)	0.25	1.01 (1)	0.53	1.01 (1)	0.53	1.01 (1)	0.49
	ETS < 2 & sleep duration 6 to 9	1 (Ref)		1 (Ref)		1 (Ref)		1 (Ref)	
	ETS < 2 & sleep duration≥10	0.88 (1)	**<0.01**	0.9 (1)	**<0.01**	0.91 (1)	**<0.01**	0.91 (1)	**0.02**

*Exponentiated value providing ratio; ETS, exposure to sunlight.

Model 1: Crude model.

Model 2: Model 1 adjusted for age and sex.

Model 3: Model 2 adjusted for physical status (obesity, prevalent hypertension, prevalent diabetes, and prevalent dyslipidemia).

Model 4: Model 3 adjusted for lifestyle status (smoking status, drinking status, and regular exercise) and socio-demographic factors (family income, educational level, occupation, and region of residence).

## Discussion

25(OH)D is known to be involved in the metabolism of calcium and phosphate for the maintenance of musculoskeletal health, but recent clinical studies have suggested it may also be involved in chronic metabolic disease. Research has shown that 25(OH)D receptors are present in most cells of the body and produce a variety of symptoms due to the hormone-like effects of 25(OH)D. Vitamin D deficiency is often overlooked because of the lack of outward symptoms. UV light exogenously triggers 25(OH)D production, and 25(OH)D can be supplemented through food intake. Differences in the bioavailability of 25(OH)D have been reported to depend on the form, route of administration, and activation pathway involved^33^. In modern society, it can be difficult to obtain adequate sun exposure because of an increase in indoor activities. Therefore, it is important to study the combined influence of low exposure to sunlight and other factors in vitamin D deficiency to indicate more achievable measures that can be taken against this condition. Our study investigated vitamin D deficiency from this angle, and in line with previous reports, we found that sun exposure and vitamin D status have a significant association. This is a natural consequence of the 25(OH)D activation pathway. However, unlike previous studies, we also analyzed the relationship between sleep duration and vitamin D status in detail. We found no significant difference between vitamin D status and sleep duration in individuals who were exposed to adequate sunlight. However, in participants with insufficient sun exposure, we found that individuals with excessive sleep duration had relatively low levels of 25(OH)D, even after controlling for potentially confounding variables.

A previous study reported that shorter sleep duration was associated with lower vitamin D status in the elderly^34^, though the data were limited by the age group of the study population and did not sufficiently control for sun exposure and other confounding factors. Another study found a relationship between sleep apnea and vitamin D status, but did not analyze other characteristics of sleep duration^35^. Although vitamin D supplements have been reported to improve sleep quality^36^, their effect on serum vitamin D status and sleep duration has not been assessed.

This study was based on a population-based epidemiologic dataset and controlled for the effect of sun exposure on vitamin D status to mimic life in a contemporary society. Results were statistically significant, even when potentially confounding variables were controlled. However, the factors such as adequate sleep duration and exposure to sunlight vary depending on the person and the given environment, so the lack of sufficient reflection of these factors may be a limitation of our research. In addition, because sleep duration and sun exposure were determined from participant responses to a questionnaire, the accuracy of these data may be limited by leading question bias or recall bias.

In this study, we found that low serum vitamin D status are associated with excessive sleep duration in individuals with low sun exposure. Therefore, in modern society where sun exposure is inevitably low, maintaining an adequate serum vitamin D status may be important for a healthy sleep duration.

## Conclusion

Therefore, we analyzed the relationship between sleep duration and vitamin D status in the low exposure to sunlight group. There was no significant difference between vitamin D status in the sleep insufficiency and normal-range sleep groups. However, the excessive sleep group had significantly lower levels of 25(OH)D than the normal-range sleep group, even after controlling for confounding variables.
